# Food Environment in and around Primary School Children’s Schools and Neighborhoods in Two Urban Settings in Kenya

**DOI:** 10.3390/ijerph18105136

**Published:** 2021-05-12

**Authors:** Constance Awuor Gewa, Agatha Christine Onyango, Rose Okoyo Opiyo, Lawrence Cheskin, Joel Gittelsohn

**Affiliations:** 1 Department of Nutrition & Food Studies, College of Health & Human Services, George Mason University, 4408 Patriot Circle, Suite 4100, MSN 1F7, Fairfax, VA 22030, USA; lcheskin@gmu.edu; 2 Department of Nutrition & Health, Maseno University, Maseno P.O. Box 333-40105, Kenya; acatieno@yahoo.com; 3 School of Public Health, University of Nairobi, Nairobi P.O. Box 30197-00100, Kenya; roseopiyo@uonbi.ac.ke; 4 Department of International Health, Bloomberg School of Public Health, John Hopkins University, Baltimore, MD 21205, USA; jgittel1@jhu.edu

**Keywords:** school children, food environment, food healthiness, urban settings, Kenya

## Abstract

We conducted a cross-sectional study to provide an overview primary school children food environment in two urban settings in Kenya. Six schools, catering to children from low-, medium- and high-income households in the cities of Nairobi and Kisumu in Kenya, participated in the study. Data on types of food places and foods offered were collected and healthy and unhealthy food availability scores calculated for each place. We utilized prevalence ratio analysis to examine associations between food availability, food place characteristics and neighborhood income levels. Altogether, 508 food places, located within 1 km of the schools and the school children’s neighborhoods were observed. Open-air market sellers and kiosks were most common. The proportion of food places with high healthy food availability was 2.2 times greater among food places in Nairobi compared to Kisumu, 1.9 times greater in food places with multiple cashpoints, 1.7 times greater in medium/large sized food places and 1.4 times greater in food places located in high income neighborhoods. These findings highlight differences in availability of healthy foods and unhealthy foods across types of food places and neighborhood income levels and inform public health interventions aimed at promoting healthy food environments in Kenya.

## 1. Introduction

Countries in the East African region are undergoing a nutrition transition with increasing prevalence of overweight and obesity among different population groups [1,2,3,4,5]. The most recent nationally-representative demographic health survey conducted in Kenya in 2014 showed that 23% of women of reproductive age were overweight while 10% were obese [1]. Studies conducted among urban-based primary school children have reported an overweight/obesity prevalence of 19–20% [6,7]. Understanding factors contributing to these changes can contribute to obesity prevention efforts. There is an increasing interest in the role of the food environment in influencing an individual’s nutritional status [8,9,10]. The food environment is defined as the interface that mediates people’s food acquisition and consumption within the wider food system that encompasses external dimensions such as the availability, prices, vendor and product properties, and promotional information; and personal dimensions such as the accessibility, affordability, convenience and desirability of food sources and products [11]. Research studies conducted in high and middle income nations have shown that one’s food environment plays a role in influencing diet quality and obesity risk [12,13,14,15]. Availability of healthy and unhealthy food options affects purchase decisions and consumption patterns among children and adults [16,17,18,19].

Despite the rising prevalence of childhood overweight and obesity in Kenya, not a single study has examined school children’s food environment in Kenya. Research on the food environment in sub-Sahara Africa is still limited. Furthermore, a majority of food environment-related research in the region has been conducted in South Africa with fewer studies conducted in other parts of the region [20]. Informal vendors still make up a large proportion of the food retailers in sub-Sahara Africa [21]. Food places like kiosks, market stalls, roadside stalls are physically accessible, allow customers to negotiate prices to some extent and may allow customers to buy food on credit, and offer a fairly large number of cereals, legumes, and fresh vegetables but relatively limited number of processed products and smaller packaging sizes [22,23]. However, the numbers of cooperate and independently-owned supermarkets and fast-food restaurant chains in sub-Sahara Africa have been on the rise [24,25]. Research conducted outside Kenya has shown that supermarkets offer a large variety of non-food and food products including fruits and vegetables, and frozen, canned and cooked foods, and offer foods at lower prices [22,23]. Additionally, a globalized market has increased the pace at which processed foods become available in Africa [26,27]. Food processing methods such as pasteurization, nutrient enrichment and food fortification have contributed to food safety, food security and improved nutrition amongst human populations [28]. However, highly-processed or ultra-processed foods are unhealthy as they contain high amounts of added sugars, saturated fats and sodium and are poor sources of health-promoting nutrients like protein, fiber, vitamins and minerals [29,30,31,32]. Consumption of highly-processed or ultra-processed foods has been associated with higher risks of obesity, cancer, metabolic syndrome, gastro-intestinal disorders, cardiovascular disease and all-cause mortality [33].

Studies conducted in the US have shown that healthful school food environment are significantly associated with lower intake of calories of sugar-sweetened beverages, low-nutrient, energy-dense foods and lower likelihood of obesity among school children [13,34]. A meta-analysis on the effectiveness of school food environment on children’s dietary behaviors showed that interventions that provided healthful foods and beverages were associated significant increase in fruit and vegetable consumption, health-oriented competitive food and beverage policies were associated with significant reductions in consumption of sugar-sweetened beverages and unhealthy snacks while more healthful policies about school meal standards were associated with significant increase in fruit consumption and reduced intake of sodium, total fats and saturated fats [35]. Hence, exposing children to a healthy food environment has the potential to contribute to preventing obesity in different populations.

The Agriculture, Nutrition and Health Academy Food Environment Working Group (ANH-FEWG) conceptual framework situates the food environment as the interface that mediates people’s food acquisition and consumption within the wider food system that encompasses external dimensions such as the availability, prices, vendor and product properties, and promotional information; and personal dimensions such as the accessibility, affordability, convenience and desirability of food sources and products” [11]. Our study addresses the availability construct found within the external domain of the ANH-FEWG conceptual framework. Though considered external to the individual, the types of foods available within a child’s food environment are likely to influence their food acquisition and consumption practices. Increased food expenditure at modern food retailers in Zambia were associated with increased diet diversity as well higher energy, mineral and vitamin intake among children [36]. A study conducted among fourth graders in South Africa found that over 80% of the students bought food from school tuck shops with energy-dense foods being most popular. Similarly, a study conducted in primary schools in Eswatini, reported that most of the offerings in school-based snack shops were energy-dense with low nutrient density [37]. Thus, food environments present potential areas for public health strategies aimed at supporting healthy lifestyles and preventing overweight and obesity.

Studies have shown that availability of healthy food options vary across multiple factors including type of vendor and neighborhood socio-economic status amongst others. Formal retailers in Ghana were predominant sources of unhealthy foods including sugary drinks, confectioneries, sugar while in South Africa, formal retailers predominantly provided a mix of healthy and unhealthy food options like highly processed meats, sugar, legumes and vegetables [38]. Studies conducted in the United States have shown that grocery stores and supermarkets offer a larger variety healthy food options compared to corner stores and convenience stores [39]. Additionally, studies conducted in high income nations have shown that low income neighborhoods have higher exposure to unhealthy food options [40]. Therefore exploring the availability of healthy and unhealthy foods across types of retailers and across neighborhood income levels would help inform interventions aimed at supporting healthy food choices in Kenya and similar countries in sub-Sahara Africa.

We conducted a study to provide an overview primary school children food environment in two urban settings in Kenya. Specifically, we examined:

(1) types of food places located around and within school settings and school children’s neighborhoods
(2) healthy/unhealthy food availability in food places located around and within school settings and within school children’s neighborhoods
(3) associations between healthy and unhealthy food availability and food place characteristics (type and size)
(4) association between healthy and unhealthy food availability and neighborhood income levels.

## 2. Materials and Methods

The food environment study was part of a research study aimed at assessing determinants of overweight and obesity among school children in Kenya. Ethical approval for the study was obtained from the Office of Research Subject Protections at George Mason University, Maseno University Ethics Review Committee the National Commission for Science, Technology and Innovation (NACOSTI). Parents, school principals and retail food sources and prepared food source managers were informed in detail about the aim and procedures of the study and parental and child assent sought prior to commencing research study activities.

### 2.1. Study Setting and Population

The obesity study utilized a cross-sectional study design and was conducted in Kisumu and Nairobi cities of Kenya in May–July 2019. The prevalence of overweight and obesity is higher among women living in urban areas of Kenya compared to those living in rural parts of the country [1]. Conducting the current study in Nairobi and Kisumu gave us the opportunity to examine childhood obesity-related factors in two of the largest cities in Kenya. The few studies that have examined prevalence of obesity among school children in Kenya have been conducted in or around Nairobi. Nairobi is the capital city of Kenya and sub-divided into eight administrative Sub-Counties with a total population of 4.4 million people [41]. Nairobi is an international, regional, national and local hub for commerce, transport, regional cooperation and economic development, and connects eastern, central and southern African countries [42]. Results from a survey conducted by the World Bank showed that 56% of the households in Nairobi city reported household expenditures above the poverty line [43]. Approximately 60% of households in Nairobi city are food insecure [44]. Kisumu city is the third largest city in Kenya and provides an opportunity to compare childhood obesity patterns across cities of different income and developmental levels. Results from a survey conducted by the World Bank showed that 51% of the households in Kisumu reported household expenditures above the poverty line [43]. Over 70% of households in Kisumu are food insecure [45].

The following process was followed in identifying schools and recruiting school children to participate in the obesity study. Lists of public primary schools were acquired from the respective local government education offices and a decision was made to identify schools located within one Sub-County in each city. Westland Sub-County in Nairobi and Kisumu Central Sub-County in Kisumu were selected for their ease of reach from the city centers. Although the Government of Kenya has implemented free primary education, there are still certain costs associated with attending public primary schools in Kenya [46]. Primary schools that predominantly cater to children from high income schools are associated with higher costs of attendance and vice-versa. We purposefully selected the most populous public primary schools catering to students from low, middle and high income households, in each Sub-County, to participate in this study. The schools catering to students from low- and high-income households were located in low and high income neighborhoods, respectively. The school catering to students from middle income households in Kisumu was located in a middle-income neighborhood while the school catering to students from middle income households in Nairobi was located in a high income neighborhood. School children’s study sample size was estimated using the formula n = z^2^ p(1 − p)/d^2^, where “z” is the critical value and in a two-tailed test = 1.96, “p” is the proportion of overweight or obese school-age children and “d” is the absolute sampling error that can be tolerated and was set at 0.05. The estimated the proportion of overweight or obese school-age children was set at 0.19 [6]. The calculated sample size was corrected to account for the finite population size using the formula n_c_ = nN/(n + (N − 1)) [47]. Sixty-five to seventy children, ages 10–12 years (grades 4–6) in each school were randomly selected to participate in the obesity research study.

We report on the participating school children’s food environment in this report. Food retail outlets and prepared food sources located within one kilometer of the schools were sampled for observation. School children’s neighborhoods were listed and the top three neighborhoods, based on percentage of study children living in the neighborhood, were included in the food environment’s sampling area. School children’s residences were not limited to Westlands and Kisumu Central Sub-Counties. Food retail outlets and prepared food sources located within one kilometer of the neighborhood shopping centers were sampled for observation. Examples of food retail outlets included supermarkets, minimarts, shops, kiosks and free-standing vendors. Prepared food sources included restaurants, food kiosks and individual vendors selling ready-to-eat, cooked or processed foods.

Descriptions of the food places have been included in Table A1. We categorized food vendors by size for the purposes of sampling. “Very small” vendors were individual market vendors and street vendors. “Small” vendors included kiosks. The most common kiosks in Kenya are semi-permanent structures often constructed of a mix iron-sheets, steel, wood and cardboards and measure about three meters square or below in area [48,49]. Similar-sized shops or restaurants were classified as “small” vendors. Medium-sized vendors included minimarts and medium sized restaurants and large vendors included supermarkets, fast-food places and large restaurants. All school-managed or within-school canteens or lunch programs at the participating schools were included in the study. All large and medium-sized establishments within each sampling area were included in the study. Sampling of small and very small food places was different. In crowded areas, defined as areas where very-small or small vendors were immediately adjacent to each other, every 5th small or very small vendor was included in the study. In non-crowded areas, all small and very small food retail outlet were included in the study. Previous studies have categorized food vendors into formal and informal market categories [27]. Some individual vendors and kiosk owners included in the current study operated their businesses at legally-sanctioned market places, and we were not able to classify them into formal/informal market categories. Four recent university graduates (two in each city) were trained to conduct the food establishment observations. Checklists, previously used in Baltimore, were adapted and modified for use in Kenya [50,51]. The food retail outlets’ checklist assessed for availability of fresh produce, processed foods, cooked foods, and drinks. Prepared food sources’ checklist assessed for availability of main dishes, staples, beverages, snacks and condiments. We updated the list of foods within each category to include foods found in Kenya. Common names (English, Kiswahili and local language) of listed foods were discussed and recorded, and adapted food lists pretested and updated prior to being used for data collection. Please see Table A2 and Table A3. Observations were conducted between 9.00 am and 5.00 pm during the weekdays and were conducted in June 2019 with observations conducted in a total of 364 food retail outlets and 144 prepared food sources for a total of 508 food places. Six of these were within school canteens or lunch programs.

### 2.2. Classifying Foods into Healthy and Unhealthy Food Categories and Food Healthines Index

We first categorized foods into food groups vegetables; fruits; legumes, nuts and seeds; meats; fish; poultry; grains; dairy; non-dairy drinks; oils and fats and condiments. Highly formulated food products like crisps were classified based on their main ingredient. We then utilized Kenya’s food-based dietary guidelines to categorize foods into healthy and unhealthy categories (Table 1). 

Kenya’s food-based dietary guidelines promote consumption of a variety of nutrient dense foods while limiting added sugars, high sodium foods and foods rich in saturated fats [52]. The use of national food-based dietary guideline or national food standards in classifying foods into healthy and unhealthy foods is recommended by the International Network for Food and Obesity/Non-communicable Diseases (NCDs) Research, Monitoring and Action Support (INFORMAS), a global network of public-interest organizations and researchers whose work focuses on food environments, obesity and non-communicable diseases [53]. We made certain assumptions to facilitate the food classification process:

- Hot ready-to-eat foods and cold salads assumed were assumed to be prepared at each vendor’s site. Food such as yogurt, ice-cream, processed juices, bread, etc. assumed to be processed at a commercial site outside vendor’s location.
- Foods were classified into food groups based on their most prominent ingredient e.g., Beef stew was classified into the meats food group.
- The Government of Kenya mandates that commercially processed flours be fortified with vitamins and minerals [54]. Hence, we classified maize flours in the healthy grain category.
- Observers did not indicate milk fat levels of milk or milk products not marked as low fat. Kenya’s dairy standards requires milk fat levels of at least 3.25% and an analysis of milk content across value chain recorded milk fat levels of 3.33–3.89%, and we made the decision to classify milk and milk products with unknown fat content as whole-fat [55].
- Regular canned fish and vegetable tend to contain higher sodium levels compared to their non-processed versions and were categorized in the unhealthy food category [56].

### 2.3. Defining Food Availability Scores

We developed a healthy food and unhealthy availability scores for the purposes of this analysis. For the healthy foods, a single point was assigned to each food group if at least one type of healthy food within the respective food group was available in the food place, and points summed up to define healthy food availability score (HFAS), with a higher HFAS indicating higher diversity of healthy food options and vice-versa. For the unhealthy foods, a single point was assigned to each food group if at least one type of unhealthy food within the respective food group was available, and points summed up to define unhealthy food availability score (UFAS), with a higher UFAS indicating higher diversity of unhealthy food options and vice-versa. The developed HFAS and UFAS were not validated against any other metric. Food availability scores have been used in previous research studies [39,57,58].

Inter-observer reliability: 159 of the 508 food places were visited by two observers to allow for inter-observer comparisons. The research team discussed any differences in observations and recordings and resolved any differences. Inter-observer reliability was further assessed by computing HFAS and UFAS based on each observer’s records of the foods available at the 159 food places. A comparison of resulting scores and indices showed very high levels of agreement with Spearman’s correlation rank values of at least 0.97.

### 2.4. Food Place Cashpoints

We categorized food places into those with one cashpoint and those with more than one cashpoint.

### 2.5. Healthy Cooking Methods

Information on the number of healthy cooking methods used to prepare foods was collected from the vendors. Healthy cooking methods included methods boiling, baking, grilling, steaming, etc. Frying was not considered as a healthy cooking method.

### 2.6. Neighborhood Income Status

We utilized previously-defined neighborhood income status in Nairobi and Kisumu, to categorize school children’s school and residential neighborhoods into low, middle and high income neighborhoods [45,59].

### 2.7. Data Analysis

Data analysis was performed using SAS version 9.4 (SAS Institute, Cary, NC, USA). Descriptive statistics were used to summarize food retail outlets and prepared food sources’ characteristics within each city. Chi-square statistics were used to compare food place characteristics and availability of specific foods/food groups across cities. The HFAS and UFAS medians and first and third quartile values were estimated. Wilcoxon-Mann-Whitney test was used to compare food availability scores/index across the two cities.

The HFAS and UFAS were each grouped by terciles to create food places with low, medium or high HFAS and UFAS. Prevalence ratio analysis was utilized to examine the association between dependent variables (high HFAS and high UFAS) and each of the following independent variable: city, retail outlets versus prepared food places, number of cash points, food place size (very small, small and medium/large) and neighborhood income level. First, we conducted univariate analysis for each independent variable and dependent variables. Each prevalence ratio (PR) reported in the bivariate analysis represents the probability of having a high HFAS or high UFAS that is associated with the respective independent variable. We then conducted separate multivariate regression analysis assessing the association between dependent variables (high HFAS and high UFAS) and (i) number of cashpoints, (ii) food place size, and (iii) neighborhood income level, while controlling for city and type of food place (retail or prepared food source). Model fitness was assessed for each regression model. Six of the observed food places were within school canteens or lunch programs and were not included in analyzed and summarized separately.

## 3. Results

### 3.1. Types of Food Places

A total of 364 food retail outlets and 144 prepared food sources were observed. Six of these were within school canteens or lunch programs. Approximately 50% of observed retail food places were open-air market sellers (Table 2). Approximately 66% of the retail food places in Kisumu were open-air market sellers compared to 21% in Nairobi. Kiosks were the most predominant prepared food sources in both cities. About 40% of the observed prepared food sources were kiosks. Only 12% of the food retail outlets had more than one cashpoint. Overall, 74%, 10% and 12% of supermarkets, minimarkets and butcheries, respectively had more than one cashpoint. The remaining food retail outlets did not have more than one cashpoint. Only 6% of prepared food sources, all of which were restaurants, had more than one cashpoint.

**Figure 1:**
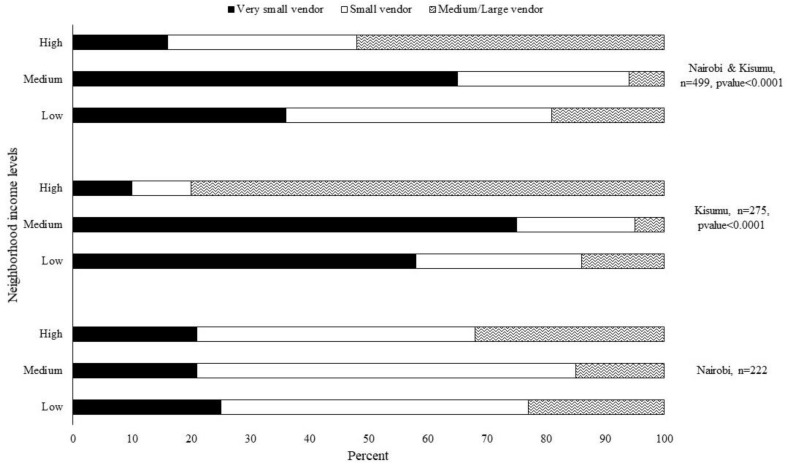
Percentage of very small, small and medium/large-sized food places in low, medium and high income neighborhoods in Nairobi and Kisumu.

Approximately 35% of the observed retail food places were located within low income neighborhoods, 50% were in medium income neighborhoods and 15% were in high income neighborhoods (Table 3). Approximately 31% of the observed prepared food sources were located within high income neighborhoods, 29% were in medium income neighborhoods and 40% were in low income neighborhoods. An examination of food places across neighborhood income levels showed that 52% of food places in high income neighborhoods were medium/large-sized compared to 19% and 6% of food places located in low and middle income neighborhoods (Figure 1). Overall, only 16% of food places in high income neighborhoods were very small-sized compared to 36% and 65% of food places located in low and middle income neighborhoods.

Sixty-three percent of the prepared food sources utilized healthy cooking methods including boiling, baking and grilling. Sixty-seven percent of the observed prepared food sources offered at least one type of healthy side including *kachumbari* (salsa), coleslaw with no mayonnaise, fruits or fruit salad, and vegetable salads.

### 3.2. Availability of Healthy and Unhealthy Foods

Approximately 54% of the food retail outlets offered healthy vegetable options. (Table 4). Over 40% offered legumes, nuts or seeds and offering eggs or skinless poultry options. About 30–39% offered fruits and recommended grain options and 29% offered healthy non-dairy drinks and snacks. Lean ground beef and low fat milk options were least common with 7% and 5% offering these options, respectively. The percentage of food retail outlets that offered healthy food options, specifically vegetables, legumes, nuts and seeds, meats, fish, poultry, recommended grains, healthy dairy, non-dairy beverages, condiments, and vegetable oils and fats was significantly higher in Nairobi compared to Kisumu. Over 60% of the prepared food sources served healthy vegetable options, poultry options and non-dairy drinks. Over 50% served recommended grains and dairy beverages. Healthy meat options were least common. The percentage of prepared food sources that served healthy food options were quite similar across the two cities. Food retail outlets’ HFAS ranged from 0 to 11 with a median of 2 (1, 5). The HFAS in Nairobi-based retail outlets were significantly higher than in Kisumu-based outlets [a median of 4 (2, 7) versus a median of 1 (1, 2), *p*-value < 0.0001]. Prepared food sources’ HFAS ranged from 0 to 9 with a median of 5 (2, 7). There was no significant between-city differences in prepared food sources’ HFAS.

For the unhealthy food options, unhealthy grain products were most common with 41% of the retail food places stocking at least one type of refined grain products commonly prepared with high levels of fat or added sugars. About 23–28% of the retail places offered deep-fried vegetable options, high fat/high sugar dairy and non-dairy options and condiments. Animal fats (butter and ghee) were least common. The percentage of food retail outlets that offered unhealthy vegetable options, grains products, dairy and non-dairy beverages, and condiments was significantly higher in Nairobi compared to Kisumu (Table 4). Sixty-nine and seventy-nine percent of the prepared food sources served unhealthy meat options and grain options, respectively. The percentage of prepared food sources that offered unhealthy poultry options and dairy products was significantly higher in Kisumu compared to Nairobi. Food retail outlets’ UFAS ranged from 0 to 9 with a median of 0 (0, 3). The UFAS in Nairobi-based retail outlets were significantly higher than in Kisumu-based outlets [median of 1 (0, 5) versus a median of 0 (0, 1), *p*-value < 0.0001]. Prepared food sources’ UFAS ranged from 0 to 6 with a median of 3 (2, 4). There was no significant between-city differences in prepared food sources’ UFAS.

### 3.3. Healthy Food Availability in Food Places Located within and Near Schools

**Figure 2:**
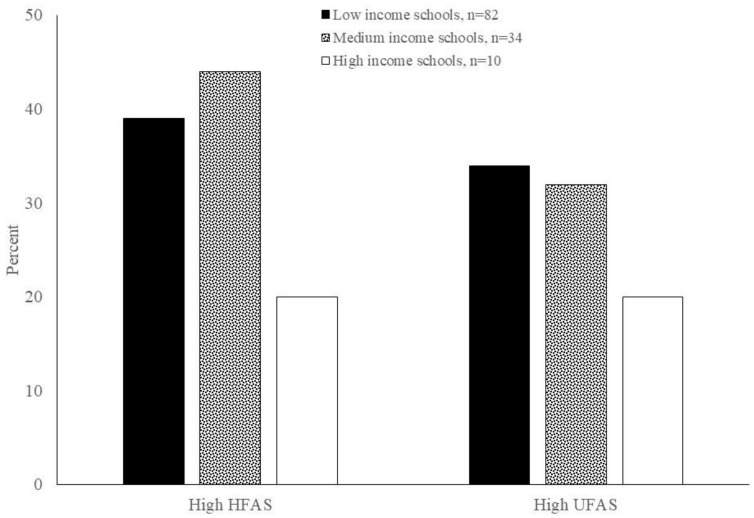
Percentage of food places within schools and schools’ immediate neighborhoods that had high healthy availability scores (HFAS) and high unhealthy availability scores (UFAS).

Our assessment of food places located within schools and schools’ immediate neighborhood revealed that the low-income schools had the highest number of food places (*n* = 82) compared to the medium- (*n* = 34) and high-income (*n* = 10) schools (Table 5). The location of the low-income school in Westlands, Sub-County in Nairobi was adjacent to a large fresh produce market. The availability of healthy foods varied across schools. Figure 2 shows places with high HFAS and high UFAS within and around the schools. Overall, 39% of the food places (retail outlets and prepared food sources) inside and around the low-income schools had high HFAS, compared to 44% of food places inside and around middle income schools and 20% of food places inside and around high-income schools. For the unhealthy foods, 34% of the food places inside and around the low-income schools had high UFAS, compared to 32% of food places inside and around middle-income schools and 20% of food places inside and around high-income schools.

### 3.4. Association between Healthy/Unhealthy Food Availability and Food Place Characteristics

Results from the regression analysis showed that the proportion of food places with high HFAS was 2.2 times greater among food places located in Nairobi compared to food places located in Kisumu (Table 6). The proportion of food places with high HFAS was 2.4 times greater among food places with more than one cashpoint compared to those with only one cashpoint with statistical significance maintained after controlling for type of outlet (retail or prepared food source) and city (multivariate model). The proportion of food places with high HFAS was five times greater among small-sized food places compared to very small-sized food places. However, statistical significance was lost after controlling for type of outlet (retail or prepared food source) and city. The proportion of food places with high HFAS was seven times greater among medium/large-sized food places compared to very small-sized food places with statistical significance maintained after controlling for type of outlet (retail or prepared food source) and city. The proportion of food places with high HFAS was 0.6 times lower among food places located in medium income neighborhoods compared to food places located in low income neighborhoods. However, statistical significance was lost after controlling for type of outlet (retail or prepared food source) and city. The proportion of food places with high HFAS was 1.5 times higher among food places located in high income neighborhoods compared to food places located in low income neighborhoods with statistical significance maintained after controlling for type of outlet (retail or prepared food source) and city.

The proportion of food places with high UFAS was 2.1 times greater among food places located in Nairobi compared to food places located in Kisumu (Table 6). The proportion of food places with high UFAS was 3.2 times greater among food places with more than one cashpoint compared to those with only one cashpoint with statistical significance maintained after controlling for type of outlet (retail or prepared food source) and city (multivariate model). The proportion of food places with high UFAS was 18 times greater among small-sized food places compared to very small-sized food places. However, statistical significance was lost after controlling for type of outlet (retail or prepared food source) and city. The proportion of food places with high UFAS was 34 times greater among medium/large-sized food places compared to very small-sized food places with statistical significance maintained after controlling for type of outlet (retail or prepared food source) and city. The proportion of food places with high UFAS was 0.7 times lower among food places located in medium income neighborhoods compared to food places located in low income neighborhoods. However, statistical significance was lost after controlling for type of outlet (retail or prepared food source) and city. The proportion of food places with high UFAS was 1.7 times higher among food places located in high income neighborhoods compared to food places located in low income neighborhoods with statistical significance maintained after controlling for type of outlet (retail or prepared food source) and city.

## 4. Discussion

This is the first study to assess school children’s food environment in Kenya. Food retail outlets and prepared food sources consisted mainly of open-air market sellers and food kiosks. This finding is similar to findings that have reported high presence of informal retail outlets in Uganda, Ghana, Zambia and South Africa [21,22,38]. The percentage of the supermarkets/minimart, retail outlets and fast-food restaurants were higher at the study sites in Nairobi compared to those in Kisumu, while open-air market vendors were predominant at the study sites in Kisumu. This pattern is a reflection of the differences economic between the cities [42,43].

For the most part, the percentage of retail outlets that offered healthy and unhealthy food options were significantly higher in Nairobi compared to Kisumu. The differences in retail outlet offerings in Kisumu and Nairobi is most likely a reflection of the economic differences between the two cities. Food environments in a rural setting in Uganda were found to offer less items compared to those in an urban setting [21]. While Kisumu is not in a rural setting, it is a smaller city with a lower economic stature compared to Nairobi [60]. Studies in low- and middle-income countries have found positive associations between level of urbanization and availability large food vendors including supermarkets, fast food restaurants and full-service restaurants [20]. Nairobi is the commercial capital of the country and the East African region, is located within reach of the agriculturally productive areas of the Kenya Highlands and offers an attractive market for processed foods as well as fresh produce from nearby farms. Furthermore, our results showed that the proportion of food places (retail outlets and prepared food sources) with high HFAS and with high UFAS was significantly higher in medium/large-sized food places and in food places with more than one cashpoint. These categories of food places predominantly consisted of supermarkets, minimarts and established restaurants and have been referred to as the formal retailers [27]. Larger stores are likely to offer a higher variety of products including healthy and unhealthy options. Analysis of food environments in Uganda, South Africa and Sweden showed that supermarkets and formal retailers offered higher variety of foods compared to other retail outlets including a larger selection of fresh fruits and vegetables [21,38].

The proportion of food places (retail outlets and prepared food sources) with high HFAS as well as those high UFAS was significantly higher in food places located within high income neighborhoods compared to food places in low income neighborhoods. These results indicate a higher diversity of healthy food options and unhealthy food options in high-income neighborhoods. This might be a reflection of the type of food places available in the high-income neighborhoods. As we have discussed in the paragraph above, large retailers and formal retailers offer a variety of healthy and unhealthy options compared to smaller retailers. Our results showed that medium and large sized food places were most prevalent in high income neighborhoods while the very small and small vendor (individual market vendors, street vendors, kiosks) were most prevalent in low- and medium-income neighborhoods. Mobile and informal vendors in Uganda and South Africa sold a limited number of fruits and vegetables per vendor compared to larger retailers [21]. Similar findings were reported in Mexico where an assessment of food environments in different neighborhoods found that low- and middle-income neighborhoods had a smaller number of supermarkets and a higher number of very small informal businesses [61]. Informal food vendors are likely to offer inexpensive caloric-dense foods such as *mandazi, chapati* and French fries. Production and sale of fried wheat products and potatoes requires minimal capital and offers income-generation opportunities to small traders, while providing affordable snack and meal options to their clientele. A recent study in Ethiopia showed that adolescents’ most concerned about financial limitations and food safety and considered sweets and fried foods as more affordable and packaged foods safe and hygienic compared to fruits and vegetables [62]. Future studies should conduct in-depth analysis of the association between neighborhood income levels and food environment. Research and policy initiatives should work with individual vendors and kiosk owners to increase availability of affordable, safe, hygienic, and healthy food options in low- and middle-income neighborhoods.

Our assessment of food places located within and near the schools showed that a higher number of food places were located near low-income schools compared to medium- and high-income schools. The percentage of within- and around-school food places that recorded high HFAS and high UFAS decreased as school income levels increased. The decrease in UFAS is in agreement with study results reported in other countries, while the decrease in HFAS is not. Studies conducted in the US and Mexico have reported that healthy foods are significantly less available in poor neighborhoods, and schools with more low-income students had more bodegas within walking distance [61,63,64]. As mentioned earlier, the low-income school in Nairobi was located near a large fresh produce open-air market and this may have skewed the results. Additionally, the number of schools that we observed is relatively small to make any conclusive statements about school income levels and healthy food availability. Studies conducted in Mauritius, South Africa and Lesotho showed that high fat and high sugar calorie-dense foods were most popular among the students [37,65,66,67,68]. Fruits were sold in none of school shops/canteens observed in Lesotho, 30% of schools in South Africa and 46% of schools in Mauritius [37,65,67]. More recently, parents and teachers of students enrolled in private secondary schools in India agreed that there is a high availability of unhealthy foods coupled with limited availability of healthy foods in school canteens [69]. Kenya’s national school meals and nutrition strategy 2017–2022 provides the basis for designing and implementing the school meals and nutrition programs in Kenya [70]. Future efforts should examine ways to operationalize the national strategy at the school level. Additionally, the school provides a central location from which conversations about the school and surrounding food retailers’ food offerings can be launched.

### 4.1. Study Strengths and Limitations

The study’s strengths include its inclusion of schools catering to students from three different levels of income, an in-depth examination of food place offerings, utilization of the national guidelines in defining healthy foods and examination of availability of healthy and unhealthy foods. However, it suffers certain limitations. First, the small number of schools and purposeful selection of participating schools may limit generalization of study results to other schools within similar income levels. Second, we utilized school and neighborhood-level income categories in our analysis, we recognize that some school children may come from households outside of the school- and neighborhood-defined income brackets. Third, the assumptions that we made to facilitate classification of foods into healthy and unhealthy categories may under-or overestimate the proportion of food within each of these food categories. Fourth, while the use of Kenya’s national dietary guidelines in identifying healthy and unhealthy foods is relevant to the local context, it may limit our ability to compare the results to studies conducted in other countries. We explored the use of frameworks based on foods’ processed level such as NOVA [71]. However, we did not have adequate information on all types of ingredients used to make dishes served at prepared food sources. Fifth, we defined food availability scores for the purposes of this analysis, and similar scores have been used in other studies [39,57,58]. However, the food availability scores were not validated against any metric. Furthermore, the food availability scores represent the number of food groups and a not the number of foods available. Such a definition is likely to bias against specialty vendors (e.g., fruit stand) that offer multiple types of foods within a specific food group. Sixth, while the current study is the first study to assess school children’s food environment in Kenya, we acknowledge that food availability does not equate to actual consumption.

### 4.2. Future Research Recommendations

We recommend that future studies should include a larger number of schools, compare locally-defined food availability scores to existing frameworks such as NOVA, conduct a sensitivity and specificity analysis to identify a scoring system that is least biased to all types of vendors, and explore other food environment characteristics such as pricing, marketing, food safety, food preferences and their association to school children’s dietary practices.

## 5. Conclusions

Findings of this study add to the emerging literature on the role of food environment in influencing health outcomes in Africa and contributes to public health efforts aimed at promoting healthy food consumption in Kenya and the region. The study provides a detailed description of the types of food vendors and food offerings available in school children’s environment in two urban settings in Kenya. Our results highlight differences across cities, and differences in availability of healthy and unhealthy foods across types of food places and neighborhood income levels. This information is useful in targeting public health interventions aimed at promoting health food environments in Kenya.

## Figures and Tables

**Table 1:** Classifying foods into healthy and unhealthy food categories.

Food Group	Examples of Healthy Foods	Examples of Unhealthy Foods
Vegetables	Raw vegetables (e.g., kales, cabbages, tomatoes, onions, carrots, beets, arrowroots potatoes, green bananas and *kachumbari/salsa*), cooked greens, baked crisps	Chips, regular crisps, chips, *bhajia, samosa,* vegetable sandwich, canned vegetables
Fruits	Fresh fruit, fruit salad, canned fruit in 100% juice, 100% fruit juice	Canned fruit not in 100% fruit juice
Legumes, nuts & seeds	Beans, lentils, raw or dry-roasted nuts, green grams (mung beans), *githeri*, *mukimo*	
Meat	Lean ground beef, tripe	Red meat, canned meat, sausage, red meat sandwich/burger
Fish	Fresh, dried, grilled or stewed fish, *omena (dagaa)*	Canned fish
Poultry	Skinless poultry, grilled or stewed poultry, eggs	Poultry sandwich
Grains	Whole wheat flour, whole wheat bread, porridge, *ugali*, brown rice, sorghum, millet, mixed grain flour, maize or maize flour, low fat popcorn, refined wheat flour	White rice, white bread, baked goods, pancakes *chapati*, *mandazi,*
Dairy	Low fat milk, tea or coffee with milk	Milk (full fat, flavored, fermented), milkshake, yoghurt, ice-cream, frozen yogurt, milkshake, pasteurized milk
Non-dairy drinks	Bottled or plain water, hot cocoa, tea or coffee without milk, sugar-free/diet soda	Regular soda, processed juice, energy drinks
Oils and fats	Vegetable oil or fats (includes margarines), olive oil, coconut oil	Butter, ghee
Condiments	Tea leaves	Sugar, ketchup, jam

**Table 2:** Types and size of food places found within one kilometer of schools and neighborhoods ^1^ (%).

Food Place	Size	Nairobi and Kisumu	Nairobi	Kisumu
Retail food outlets:		*n* = 364	*n* = 143	*n* = 221
Supermarket	Large	9	13	6
Mini market	Medium	2	3	1
Small shop	Small	12	19	8
Cereal shop	Small	6	15	1
Kiosk	Small	6	9	3
Butcher	Small	10	14	7
Open-air market seller	Very small	48	21	66
Temporary stand/Street vendor	Very small	7	6	8
Prepared food sources:		*n* = 144	*n* = 84	*n* = 60
Fast food (chain)	Large	8	11	3
Restaurant	Large	22	18	28
Take out	Large	13	6	22
Food kiosk	Small	40	46	32
Street vendor	Very small	14	16	12

^1^ Excludes within-school canteens/lunch programs.

**Table 3:** Characteristics of food places near schools and neighborhoods ^1^ (%).

Food place and Characteristics	Nairobi and Kisumu	Nairobi	Kisumu
Food retail outlets:	*n* = 362	*n* = 143	*n* = 219
Low income neighborhood	35	54	23
Medium-income neighborhood	50	18	70
High income neighborhood	15	27	7
Having multiple cashpoints	12	13	11
Prepared food sources:	*n* = 144	*n* = 84	*n* = 60
Low income neighborhood	40	57	14
Medium-income neighborhood	29	20	43
High income neighborhood	31	23	43
Having multiple cashpoints	6	3	11
Variety of healthy cooking methods:			
0	37	27	51
1–2	62	73	47
3–5	1	0	2
Variety of healthy sides:			
0	33	31	37
1–2	62	65	58
3–5	3	1	5
Unknown	2	3	0

^1^ Excludes within-school canteens/lunch programs.

**Table 4:** Healthy and unhealthy food availability in food places near schools and neighborhoods (%) ^1,2^.

Food Group	Food Retail Outlets			Prepared Food Sources		
	Nairobi & Kisumu, *n* = 362	Nairobi, *n* = 143	Kisumu, *n* = 219	Nairobi and Kisumu, *n* = 138	Nairobi, *n* = 81	Kisumu, *n* = 57
Healthy foods:						
Vegetables	54	67	45 ****	74	73	77
Fruits	39	37	41	40	37	45
Legumes, nuts & seeds	41	57	31 ****	48	42	55
Meat	7	15	1 ****	35	32	39
Fish	16	27	8 ****	43	38	50
Poultry	42	69	24 ****	64	64	63
Grains	37	50	28 ***	59	60	57
Dairy	5	4	6	52	54	50
Non-dairy drinks	29	48	17 ****	70	73	66
Oils and fats	27	44	16 ****			
Condiments	24	36	16 ****			
Unhealthy foods:						
Vegetables	23	40	13 ****	54	48	63
Fruits	4	6	0 **			
Legumes, nuts & seeds						
Meat	8	10	6	69	72	66
Fish	2	0	4 *			
Poultry				15	7	25 **
Grains	41	59	29 ****	79	79	79
Dairy	25	38	16 ****	14	9	21 *
Non-dairy drinks	28	44	17 ****	47	46	48
Oils and fats	4	1	6 *			
Condiments	27	43	16 ****			

^1^ Excludes within-school canteens/lunch programs. ^2^ Chi-square test utilized to compare between-city percentages unless indicated otherwise: * *p* < 0.05, ** *p* < 0.01, *** *p* < 0.001, **** *p* < 0.0001.

**Table 5:** Types of food sources located within schools and schools’ immediate neighborhoods (%).

Size of Food Place	Examples	Low SES Schools, *n* = 82	Medium SES Schools, *n* = 34	High SES Schools, *n* = 10
Very small	Open-air market stands, temporary stands and street vendors	30	29	40
Small	Small shops, kiosks and butcheries	47	53	30
Medium and large	Supermarkets, minimarts, and fast food places and restaurants	22	12	0
School canteens and lunch programs		1	6	30

**Table 6:** Association between food place characteristics and high healthy/unhealthy food availability score ^1,2,3,4^.

Food Availability Score and Independent Variables	Bivariate Models, *n* = 499		Multivariate Models, *n* = 499	
	PR	CI	PR	CI
Healthy Food Availability Score (HFAS)				
Nairobi (ref = Kisumu)	**2.21**	**(1.73, 2.84)**		
Retail outlet (ref = prepared food source)	0.87	(0.68, 1.12)		
Having multiple cashpoints (ref= having one cashpoint)	**2.44**	**(1.98, 3.00)**	**1.95**	**(1.54, 2.48)**
Small-sized food places (ref = very small)	**5.06**	**(3.32, 7.74)**	1.11	(0.88, 1.41)
Medium or large-sized food places (ref = very small)	**7.06**	**(4.65, 10.7)**	**1.67**	**(1.35, 2.07)**
Medium income neighborhood (ref = low income neighborhood)	**0.60**	**(0.45, 0.81)**	0.84	(0.60, 1.18)
High income neighborhood (ref = low income neighborhood)	**1.47**	**(1.15, 1.89)**	**1.42**	**(1.11, 1.81)**
Unhealthy Food Availability Score (UFAS)				
Nairobi (ref = Kisumu)	**2.14**	**(1.63, 2.82)**		
Retail outlet (ref = prepared food source)	0.87	(0.66, 1.15)		
Having multiple cashpoints (ref = having one cashpoint)	**3.24**	**(2.64, 3.98)**	**2.32**	**(1.85, 2.90)**
Small-sized food places (ref = very small)	**18.8**	**(7.75, 45.4)**	1.17	(0.95, 1.40)
Medium or large-sized food places (ref = very small)	**34.6**	**(14.5, 83.0)**	**17.6**	**(7.25, 42.8)**
Medium income neighborhood (ref = low income neighborhood)	**0.69**	**(0.49, 0.97)**	0.96	(0.65, 1.42)
High income neighborhood (ref = low income neighborhood)	**1.87**	**(1.41, 2.47)**	**1.81**	**(1.38, 2.38)**

CI: confidence interval; PR: prevalence ratio. ^1^ Excludes within-school canteens/lunch programs. ^2^ Bivariate models: PR and associated CI represents results from bivariate GEE model for each independent variable: city (Nairobi versus Kisumu), food place indicator (retail outlet versus prepared food source), number of cashpoints indicator, food place size and neighborhood income indicator. ^3^ Multivariate models: PR and associated CI represents results from multivariate GEE models that include factor (number of cashpoints indicator, food place size or neighborhood income indicator), and food place indicator (retail outlet versus prepared food source) and city. ^4^ Bolded PR and CI are statistically significant associations.

## Data Availability

The data presented in this study are available on request from the corresponding author. The data are not publicly available due to privacy.

## Appendix A

**Table A1:** Food place description.

Food Place	Food Place Description
Supermarket	Large modern self-service stores with offering a large variety of food and non-food products with multiple cash tills. Examples of supermarkets at time of study included Tuskys, Naivas, Carrefour, Khetias, Choppies and Chandarana
Mini market	Similar to supermarkets but relatively smaller in size and with a limited variety of products and cash tills
Small shop	Modern food retail outlet housed in a permanent building that offers over-the-counter service. Small in size and sells fewer products compared to minimart.
Cereal shop	Either a small shop or kiosk that predominantly sells dry cereals e.g., maize, beans, peas, etc.
Kiosk	Most common kiosks in Kenya are semi-permanent structures often constructed from a mix iron-sheets and measure an area of about three meters square or below. Kiosks offer a limited number of raw, commercially processed or hot foods.
Butcher shop	Specialized shop that predominantly sells meats. Most butcher shops are similar to kiosks and small shops in size. May be semi-permanent structure or located in a permanent building
Open-air market seller	Stalls or vendors positioned in a legally-sanctioned open-air market place. Usually sell s, usually with a small number of wares
Temporary stand/Street vendor	Stall or vendors positioned on streets and side walks
Fast food (chain)	Modern quick service restaurants with limited dine-in or table service. Mostly located within big shopping malls. Part of restaurant chains e.g., KFC,
Take-out	Quick service restaurants with limited dine-in or table service, not part of a restaurant-chain
Restaurant	Modern restaurants that offer predominantly dine-in or table service

**Table A2:** Food retail outlets’ checklist.

Proteins, Breads and Sides	Snacks and Breakfast	Condiments	Dairy	Fresh Produce
Fish: fresh, canned	Regular crisps	White sugar	Low fat milk	Fresh vegetables varieties
Dried fish	Baked crisps	Artificial sweetener	Full cream	Root vegetables varieties
Processed or canned meat	Chips (fries)	Ketchup	Raw milk	Starchy tubers varieties
Lean ground meat	Raw/dry-roasted nuts	Tea leaves	Pasteurized milk	Fresh fruits
Skinless poultry	Bhajias	Low fat/low calorie condiments	Fermented milk	Fruit salad
Eggs	Mandazi	Jam	Powdered milk	
Dry beans	High fiber breakfast cereals		Flavored milk	Cooked foods
Dry lentils	Low sugar breakfast cereals	**Oils and fats**	Milkshake	Cooked vegetables
Maize or maize flour	Low fat popcorn	Vegetable oil	Yogurt	Cooked tubers
Refined wheat flour	Instant noodles	Olive oil	Butter	Hot coffee or tea
Whole wheat flour	Cake	Coconut oil	Ghee	
White rice	Crackers		Powdered milk	
Brown rice	Sausages	Non-dairy beverages	Cheese	
Sorghum grains or flour	Samosas	Bottled water		
Millet grains or flour		100% fruit juice		
Mixed whole grain flour	Canned fruit	Soda (regular)		
Bread (refined grain)	Canned fruit (in 100% juice)	Sugar free soda		
100% whole wheat bread	Canned fruit (not in 100% juice)	Processed juice		
Canned vegetables		Energy drinks		
Cooking banana		Hot cocoa		

**Table A3:** Prepared food places’ checklist.

Stew, Meat or Greens:	Staples:	Desserts, Drinks or Snacks:
Grilled or stewed red meat	Ugali	Baked goods
Red meat sandwich/burger	Stewed/boiled bananas	Fruit without sugar (includes fruit cups)
Matumbo (tripe)	Chapatis/rotis	100% fruit juice
Grilled or stewed poultry	White rice	Fresh fruit smoothie
Poultry sandwich or burger	Brown rice	Yogurt
Grilled or stewed fish	Chips	Fermented milk (maziwa lala)
Dagaa/Omena		Frozen yogurt
Egg, fried	Accompaniments:	Ice cream
Egg, boiled	Bread (refined grain)	Milkshake
Ndengu	100% whole wheat bread	Energy drinks
Beans	Pancakes	Regular soda
Githeri	Mandazi	Diet soda
Mukimo	Samosa	Water
Cooked greens	Porridge (non-refined grains)	Low fat milk
Non-fried vegetables	Arrowroot, sweet potato	Flavored milk
Vegetarian sandwich	Avocado	Tea or coffee with milk
		Tea or coffee without milk
	Healthy sides:	Raw or dry-roasted nuts
	Kachumbari	Crisps, regular
	Cole slaw (no mayo)	Crisps, baked
	Salad	
	Fruits	
	Vegetables	
